# The physiologic response to epinephrine and pediatric cardiopulmonary resuscitation outcomes

**DOI:** 10.1186/s13054-023-04399-5

**Published:** 2023-03-13

**Authors:** Ryan W. Morgan, Robert A. Berg, Ron W. Reeder, Todd C. Carpenter, Deborah Franzon, Aisha H. Frazier, Kathryn Graham, Kathleen L. Meert, Vinay M. Nadkarni, Maryam Y. Naim, Bradley Tilford, Heather A. Wolfe, Andrew R. Yates, Robert M. Sutton, the ICU-RESUS and the Eunice Kennedy Shriver National Institute of Child Health and Human Development Collaborative Pediatric Critical Care Research Network Investigator Groups, Tageldin Ahmed, Michael J. Bell, Robert Bishop, Matthew Bochkoris, Candice Burns, Joseph A. Carcillo, J. Michael Dean, J. Wesley Diddle, Myke Federman, Richard Fernandez, Ericka L. Fink, Stuart H. Friess, Mark Hall, David A. Hehir, Christopher M. Horvat, Leanna L. Huard, Tensing Maa, Arushi Manga, Patrick S. McQuillen, Peter M. Mourani, Daniel Notterman, Murray M. Pollack, Anil Sapru, Carleen Schneiter, Matthew P. Sharron, Neeraj Srivastava, Sarah Tabbutt, Shirley Viteri, David Wessel, Andrew R. Yates, Athena F. Zuppa

**Affiliations:** 1 Department of Anesthesiology and Critical Care Medicine, The Children’s Hospital of Philadelphia, University of Pennsylvania, 3401 Civic Center Boulevard, Wood Building – 6104, Philadelphia, PA 19104 USA; 2 Department of Pediatrics, University of Utah, Salt Lake City, UT USA; 3 Department of Pediatrics, University of Colorado School of Medicine and Children’s Hospital Colorado, Aurora, CO USA; 4 Department of Pediatrics, Benioff Children’s Hospital, University of California, San Francisco, San Francisco, CA USA; 5 Nemours Cardiac Center, Nemours/Alfred I. duPont Hospital for Children, Wilmington, DE USA; 6 Department of Pediatrics, Sidney Kimmel Medical College, Thomas Jefferson University, Philadelphia, PA USA; 7 Department of Pediatrics, Children’s Hospital of Michigan, Central Michigan University, Detroit, MI USA; 8 Department of Pediatrics, Nationwide Children’s Hospital, The Ohio State University, Columbus, OH USA

**Keywords:** Cardiac arrest, Cardiopulmonary resuscitation, Epinephrine, Adrenaline, Blood pressure, Pediatrics

## Abstract

**Background:**

Epinephrine is provided during cardiopulmonary resuscitation (CPR) to increase systemic vascular resistance and generate higher diastolic blood pressure (DBP) to improve coronary perfusion and attain return of spontaneous circulation (ROSC). The DBP response to epinephrine during pediatric CPR and its association with outcomes have not been well described. Thus, the objective of this study was to measure the association between change in DBP after epinephrine administration during CPR and ROSC.

**Methods:**

This was a prospective multicenter study of children receiving ≥ 1 min of CPR with ≥ 1 dose of epinephrine and evaluable invasive arterial BP data in the 18 ICUs of the ICU-RESUS trial (NCT02837497). Blood pressure waveforms underwent compression-by-compression quantitative analysis. The mean DBP before first epinephrine dose was compared to mean DBP two minutes post-epinephrine. Patients with ≥ 5 mmHg increase in DBP were characterized as “responders.”

**Results:**

Among 147 patients meeting inclusion criteria, 66 (45%) were characterized as responders and 81 (55%) were non-responders. The mean increase in DBP with epinephrine was 4.4 [− 1.9, 11.5] mmHg (responders: 13.6 [7.5, 29.3] mmHg versus non-responders: − 1.5 [− 5.0, 1.5] mmHg; *p* < 0.001). After controlling for a priori selected covariates, epinephrine response was associated with ROSC (aRR 1.60 [1.21, 2.12]; *p* = 0.001). Sensitivity analyses identified similar associations between DBP response thresholds of ≥ 10, 15, and 20 mmHg and ROSC; DBP responses of ≥ 10 and ≥ 15 mmHg were associated with higher aRR of survival to hospital discharge and survival with favorable neurologic outcome (Pediatric Cerebral Performance Category score of 1–3 or no worsening from baseline).

**Conclusions:**

The change in DBP following epinephrine administration during pediatric in-hospital CPR was associated with return of spontaneous circulation.

**Supplementary Information:**

The online version contains supplementary material available at 10.1186/s13054-023-04399-5.

## Background

During cardiopulmonary resuscitation (CPR), higher coronary perfusion pressure (CoPP) and greater myocardial blood flow are associated with a higher likelihood of return of spontaneous circulation (ROSC) and survival [1, 2]. In children with in-hospital cardiac arrest (IHCA), a multicenter study established an association between invasively measured diastolic blood pressure (DBP), the upstream pressure of CoPP, and survival outcomes [3]. Thus, epinephrine is recommended during CPR to augment systemic vascular resistance, thereby increasing DBP and CoPP to improve the likelihood of ROSC and survival [4].

Epinephrine is uniformly included in pediatric and adult cardiac arrest algorithms with a recommended administration frequency of every three to five minutes [4, 5]. However, clinical studies have demonstrated variable associations between epinephrine administration and patient outcomes [6, 7], suggesting that epinephrine may be beneficial in some patients during cardiac arrest but potentially not in others. In large animal studies, the physiologic response to epinephrine varies between animals and over time [8–12] and more robust increases in DBP after the first epinephrine administration are associated with higher rates of ROSC [11]. Clinically, pediatric IHCA patients vary widely in terms of demographics, arrest etiology, and other clinical characteristics [13]. In light of this clinical heterogeneity and the considerable interindividual variability in the experimental response to exogenous catecholamines [14–17], we hypothesize that the physiologic response to epinephrine during CPR varies between patients. However, the physiologic response to epinephrine during CPR has not been well described clinically and the association between this response and IHCA outcomes is unknown.

To address this knowledge gap, we leveraged data from a prospective, multicenter, cluster-randomized interventional trial (The ICU-RESUScitation Project [ICU-RESUS]; NCT02837497) in children with IHCA [18]. Our objectives were to describe the change in DBP after the first dose of epinephrine during pediatric CPR and to determine the association of this change in DBP with attaining ROSC.

## Methods

### Study setting and oversight

The ICU-RESUS study was a multicenter, hybrid stepped-wedge cluster-randomized trial of a quality improvement bundle of physiology-directed bedside CPR training and structured post-arrest debriefing [18, 19]. It was conducted in 18 pediatric intensive care units (PICUs) and pediatric cardiac intensive care units (CICUs) in the USA. The institutional review boards of each clinical site and of the Data Coordinating Center (DCC) at the University of Utah approved the ICU-RESUS study protocol with waiver of informed consent.

This secondary study was designed during ICU-RESUS patient enrollment without prior examination of the data. Only data prospectively collected per the ICU-RESUS protocol were included and analyzed.

### Patient population

The ICU-RESUS study enrolled patients who were ≤ 18 years of age and ≥ 37 weeks post-gestational age who received chest compressions for IHCA in any participating ICU. Subjects were excluded if, prior to the arrest, they: (1) were not expected to survive the hospitalization due to a terminal illness or had a documented lack of commitment to aggressive ICU therapies; (2) were declared dead by neurologic criteria; or (3) had an out-of-hospital cardiac arrest associated with the current hospitalization. For this secondary observational study, only index IHCA events for a given hospitalization were included. Subjects were required to: (1) have an invasive arterial catheter in place at the time of CPR; (2) receive at least one dose of epinephrine during CPR; and (3) have evaluable DBP data from both the minute prior to and the two minutes following the first dose of epinephrine. Subjects were excluded if the quality of the arterial blood pressure waveform was insufficient to identify stops and starts in CPR or to determine DBP values.

### Data collection

Trained research coordinators at each study site collected standardized patient and IHCA data elements [20, 21], including the timing of the first dose of epinephrine to the nearest minute, as recorded by the clinical team. Bedside monitor waveform data were captured by IntelliVue Information Center iX (Philips, Andover, MA), BedMaster (Excel Medical, Jupiter, FL), or locally developed waveform acquisition systems with acquisition rates ranging from 50 to 250 data points per second. The first ten minutes of each CPR event was locally downloaded, deidentified, transmitted to the University of Utah DCC, and then transmitted to investigators at the Children’s Hospital of Philadelphia (CHOP), who reconstructed the waveforms into an analyzable format using a custom code (MATLAB, The Mathworks, Inc., Natick, MA). At three sites without the ability to obtain fully electronic waveform data, research staff either printed waveform data from the local central monitoring system or acquired digital screenshots of the data, which were then manually digitized (PlotDigitizer; Version 2.0; Department of Physics, University of South Alabama) into the same analyzable format as the other waveforms.

### Physiologic waveform analyses

Investigators (RWM, KG, RMS) at CHOP reviewed waveforms and annotated: 1) starts and stops in CPR; 2) sections of non-analyzable arterial BP data; and 3) periods of non-sustained ROSC. For each individual chest compression, custom MATLAB code measured systolic BP (SBP) as the peak of the arterial pressure waveform and DBP as the average of data points occurring between 60 and 70% of the peak-to-peak cycle (mid-to-late diastole) as previously described [18]. This method of DBP measurement targets mid-to-late diastole to avoid incorporation of peri-compression artifact observed in some waveforms and uses multiple data points from the high-frequency data signal to further reduce the impact of spurious values. Annotations from the clinical review process were incorporated into this code to ensure that only periods of CPR were included. Events were divided into 30-s epochs, and the average SBP and DBP values for each epoch were summarized. Epochs were considered evaluable if they had at least 7.5 s of CPR data, excluding periods of non-analyzable data or intermittent ROSC. Though periods of non-sustained ROSC were excluded from all BP calculations, any epoch with more than five seconds of non-sustained ROSC was excluded from this analysis to avoid capturing periods of ROSC and potentially biasing toward higher BP calculations.

**Fig. 1:**
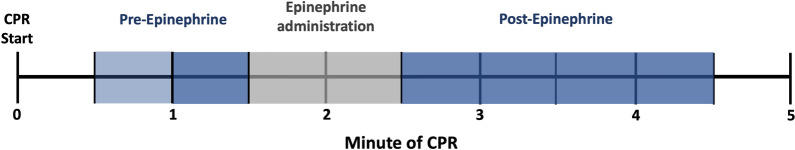
Timing of blood pressure sampling. Figure depicts timing of blood pressure sampling relative to epinephrine administration. For this theoretical patient who received epinephrine two minutes into CPR, the 30-s data epochs of that minute of CPR (e.g., 90 s through 150 s) are considered the epinephrine administration period and not included in blood pressure analyses. The immediately preceding 30-s epoch (e.g., 60 s through 90 s) is utilized for pre-epinephrine BP determination. If unavailable, the prior is used (e.g., 30 s through 60 s). The mean BP from the four post-epinephrine epochs following the epinephrine administration period (e.g., minutes 2.5 through 4.5) are utilized for post-epinephrine BP determination. The difference between the mean DBP from this 2-min period and the mean DBP from the pre-epinephrine epoch was used to classify patients as epinephrine responders or non-responders

The two 30-s epochs of the minute in which epinephrine was administered were considered the time of the epinephrine dose and were not included in analyses to avoid misclassification of pre- and post-epinephrine epochs. The 30-s epoch immediately preceding those two epochs was used to determine the pre-epinephrine BP. If not evaluable, the epoch preceding that epoch was utilized. If neither were available, the event was excluded. The four epochs following the two epinephrine epochs were considered the post-epinephrine epochs. At least one epoch in this 2-min period was required to be evaluable or the event was excluded. The average BP among evaluable epochs from this two-minute period was considered the post-epinephrine BP. For events meeting inclusion criteria with evaluable pre- and post-epinephrine BP data, the differences in DBP and SBP between the pre- and post-epinephrine were calculated (Fig. 1).

### Outcomes and statistical analysis

The primary exposure for the primary analyses was whether the patient had a DBP increase of ≥ 5 mmHg in response to the first dose of epinephrine. These patients were characterized as “epinephrine responders” and patients with < 5 mmHg rise in DBP were considered “epinephrine non-responders.” A threshold change in DBP of 5 mmHg was chosen a priori because: 1) it is likely clinically relevant in terms of the relationship between DBP and event outcomes [3, 11, 22] and 2) the investigators hypothesized that it was reasonable for bedside clinicians to be able to discern such a change during CPR. The primary outcome was sustained ROSC ≥ 20 min [21]. Exploratory outcomes included survival to hospital discharge; survival to discharge with favorable neurologic outcome, defined as Pediatric Cerebral Performance Category (PCPC) score of 1–3 (no more than moderate disability) or no worsening of PCPC from baseline; change in functional status score (FSS) of survivors from baseline to hospital discharge; and new FSS-defined morbidity [23–25].

Patient and event characteristics were summarized according to group (epinephrine responders versus non-responders) and outcome (ROSC versus no ROSC). Statistics were reported as frequencies and percentages or the median and quartiles. Outcomes were similarly summarized by group. Associations between groups and between patients with and without ROSC were examined using Fisher’s exact test for categorical variables and the Wilcoxon rank-sum test for ordinal variables.

A Poisson regression model with robust error estimates assessed the relationship between epinephrine responder status and ROSC. This model included a priori covariates hypothesized to be associated with both the DBP response and ROSC: initial CPR rhythm (bradycardia and poor perfusion versus pulseless rhythms); illness category (medical cardiac, medical non-cardiac, surgical cardiac, surgical non-cardiac); presence of a vasopressor infusion at the start of CPR; and preexisting pulmonary hypertension. A sensitivity analysis using the same model examined the associations between other potential thresholds for the change in DBP in response to epinephrine and outcomes.

Average systolic and diastolic blood pressures were graphically plotted over time relative to the time of first epinephrine dose and independently displayed for epinephrine responders and non-responders as well as for patients with and without ROSC. To further characterize the relationship between the DBP response to epinephrine and ROSC, a spline curve was generated based on a logistic regression model controlling for the same covariates as above. Euclidean distance on a receiver operating characteristic curve was minimized to determine the optimal cut point for change in DBP to discriminate patients with and without ROSC.

All analyses were performed with SAS version 9.4 (SAS Institute, Inc., Cary, NC) and two-sided p-values < 0.05 were considered statistically significant.

## Results

Of 894 ICU-RESUS patients who received epinephrine during CPR, 356 patients had some amount of evaluable BP data and 147 met all inclusion criteria and were included in the final cohort. Additional file 1: Table S1 and Additional file 2: Table S2 compare patients included in the final cohort to those who received epinephrine but did not meet inclusion criteria. In the 1186 30-s hemodynamic data epochs included, the average duration of evaluable CPR data per epoch was 28.6 ± 4.1 s. The change (median [25th percentile, 75th percentile]) in DBP with epinephrine administration was 4.4 [− 1.9, 11.5] mmHg. Sixty-six (45%) patients had a ≥ 5 mmHg increase in DBP and were classified as epinephrine responders.

**Fig. 2:**
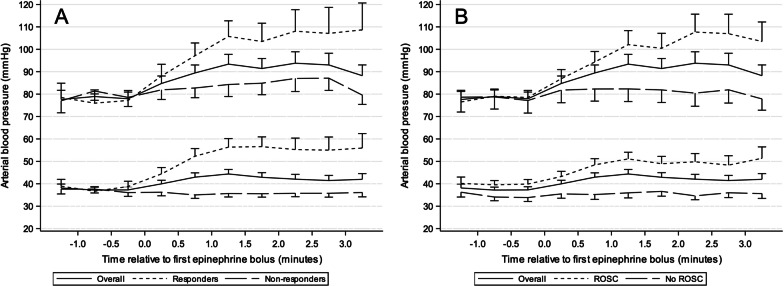
Temporal change in intra-arrest blood pressure relative to epinephrine administration. Average systolic and diastolic blood pressures plotted over time (minutes) relative to the time of first dose of epinephrine (minute 0). **A** Depicts epinephrine responders versus non-responders and **B** depicts patients with ROSC (return of spontaneous circulation) versus patients without ROSC. Each data point represents the mean value for 30-s data epochs for each patient and then averaged within each group. Error bars indicate standard error of the mean for each time point

Patient demographics and characteristics are described in Table 1. Compared with non-responders, epinephrine responders were older (*p* < 0.001); more frequently had underlying respiratory insufficiency (*p* = 0.043), pneumonia (*p* = 0.010), and a non-cardiac primary illness category (*p* = 0.005); and less frequently had congenital heart disease (*p* = 0.028). Cardiac arrest characteristics are described in Table 2. Compared to epinephrine non-responders, epinephrine responders were more frequently treated in a PICU than a CICU (*p* < 0.001), had shorter durations of CPR (*p* < 0.001), received the first dose of epinephrine earlier during CPR (*p* = 0.028), received fewer total doses of epinephrine during CPR (*p* < 0.001), and were less likely to receive calcium (*p* = 0.020) or sodium bicarbonate (*p* = 0.004) during CPR. The pre-epinephrine DBP (*p* = 0.326) and SBP (*p* = 0.327) did not differ between epinephrine responders and non-responders. The median change in DBP among epinephrine responders was 13.6 [7.5, 29.3] mmHg versus − 1.5 [− 5.0, 1.5] mmHg in epinephrine non-responders (*p* < 0.001). The median change in SBP between these groups was 24.0 [11.5, 38.3] mmHg versus 1.4 [− 9.7, 14.1] mmHg, respectively (*p* < 0.001). The values of DBP and SBP relative to the administration of the first dose of epinephrine are depicted in Fig. 2.

**Table 1:** Patient characteristics by epinephrine response

	Overall (n = 147)	Epinephrine Responders (n = 66)	Epinephrine Non-Responders (n = 81)	*p*
Demographics				
Age (years)	0.3 [0.0,1.7]	0.5 [0.1,3.3]	0.2 [0.0,0.5]	< .001
Age				< .001
< 1 month	48 (32.7%)	13 (19.7%)	35 (43.2%)	
1 month- < 1 year	57 (38.8%)	28 (42.4%)	29 (35.8%)	
1 year- < 12 years	31 (21.1%)	15 (22.7%)	16 (19.8%)	
> 12 years	11 (7.5%)	10 (15.2%)	1 (1.2%)	
Male	71 (48.3%)	31 (47.0%)	40 (49.4%)	0.868
Race				0.840
White	73 (49.7%)	33 (50.0%)	40 (49.4%)	
Black or African American	31 (21.1%)	16 (24.2%)	15 (18.5%)	
Other	10 (6.8%)	5 (7.6%)	5 (6.2%)	
Unknown or not reported	33 (22.4%)	12 (18.2%)	21 (25.9%)	
Preexisting conditions				
Respiratory insufficiency	123 (83.7%)	60 (90.9%)	63 (77.8%)	0.043
Hypotension	113 (76.9%)	52 (78.8%)	61 (75.3%)	0.696
Congenital heart disease	105 (71.4%)	41 (62.1%)	64 (79.0%)	0.028
Pulmonary hypertension	24 (16.3%)	14 (21.2%)	10 (12.3%)	0.180
Sepsis	18 (12.2%)	9 (13.6%)	9 (11.1%)	0.801
Renal insufficiency	15 (10.2%)	8 (12.1%)	7 (8.6%)	0.587
Congestive heart failure	14 (9.5%)	7 (10.6%)	7 (8.6%)	0.781
Pneumonia	14 (9.5%)	11 (16.7%)	3 (3.7%)	0.010
Malignancy	7 (4.8%)	5 (7.6%)	2 (2.5%)	0.244
Trauma	1 (0.7%)	1 (1.5%)	0 (0.0%)	0.449
Pre-event characteristics				
Illness category				0.005
Medical cardiac	38 (25.9%)	11 (16.7%)	27 (33.3%)	
Surgical cardiac	69 (46.9%)	29 (43.9%)	40 (49.4%)	
Non-cardiac	40 (27.2%)	26 (39.4%)	14 (17.3%)	
Baseline PCPC score*				0.445
1—Normal	103 (70.1%)	44 (66.7%)	59 (72.8%)	
2—Mild disability	28 (19.0%)	15 (22.7%)	13 (16.0%)	
3—Moderate disability	9 (6.1%)	2 (3.0%)	7 (8.6%)	
4—Severe disability	7 (4.8%)	5 (7.6%)	2 (2.5%)	
Baseline FSS*	6.0 [6.0,8.0]	6.0 [6.0,8.0]	6.0 [6.0,8.0]	0.413
PRISM^†^	7.0 [2.0,12.0]	7.0 [2.0,11.0]	7.0 [3.0,12.0]	0.772
Vasoactive inotropic score^‡^	4.0 [0.0,10.0]	3.0 [0.0,8.0]	5.0 [0.0,10.0]	0.300
Vasopressors used^‡^				
Dopamine	30 (20.4%)	10 (15.2%)	20 (24.7%)	0.217
Dobutamine	1 (0.7%)	0 (0.0%)	1 (1.2%)	1.000
Nitroprusside	3 (2.0%)	1 (1.5%)	2 (2.5%)	1.000
Milrinone	48 (32.7%)	17 (25.8%)	31 (38.3%)	0.116
Epinephrine	55 (37.4%)	23 (34.8%)	32 (39.5%)	0.610
Norepinephrine	6 (4.1%)	4 (6.1%)	2 (2.5%)	0.409
Vasopressin	6 (4.1%)	4 (6.1%)	2 (2.5%)	0.409

*PRISM* Pediatric RISk of Mortality, *PCPC* Pediatric Cerebral Performance Category, *FSS* Functional Status Scale

^*^Baseline PCPC and FSS represent subject status prior to the event leading to hospitalization

^†^PRISM was evaluated 2–6 h prior to the event

^‡^Vasoactive inotropic score and vasopressors used were evaluated 2 h prior to the event

Epinephrine responders (patients with ≥ 5 mmHg increase in DBP following the first dose of epinephrine administered during cardiopulmonary resuscitation) and epinephrine non-responders compared using Fisher’s exact test for categorical data and Wilcoxon rank-sum test for continuous data

**Table 2:** Cardiac arrest event characteristics by epinephrine response

	Overall (*n* = 147)	Epinephrine responders (*n* = 66)	Epinephrine non-responders (*n* = 81)	*P*
Location of CPR event				< 0.001
PICU	46 (31.3%)	31 (47.0%)	15 (18.5%)	
CICU	101 (68.7%)	35 (53.0%)	66 (81.5%)	
Interventions in place				
Central venous catheter	119 (81.0%)	52 (78.8%)	67 (82.7%)	0.673
Vasoactive infusion	102 (69.4%)	45 (68.2%)	57 (70.4%)	0.858
Invasive mechanical ventilation	120 (81.6%)	53 (80.3%)	67 (82.7%)	0.831
Non-invasive ventilation	15 (10.2%)	8 (12.1%)	7 (8.6%)	0.587
Immediate cause(s) of arrest				
Arrhythmia	23 (15.6%)	10 (15.2%)	13 (16.0%)	1.000
Cyanosis without respiratory decompensation	7 (4.8%)	3 (4.5%)	4 (4.9%)	1.000
Hypotension	101 (68.7%)	46 (69.7%)	55 (67.9%)	0.859
Respiratory decompensation	67 (45.6%)	31 (47.0%)	36 (44.4%)	0.868
Timing of CPR event*				0.150
Weekday	90 (61.2%)	46 (69.7%)	44 (54.3%)	
Weeknight	32 (21.8%)	12 (18.2%)	20 (24.7%)	
Weekend	25 (17.0%)	8 (12.1%)	17 (21.0%)	
First documented rhythm				0.706
Asystole/PEA	51 (34.7%)	25 (37.9%)	26 (32.1%)	
VF/pulseless VT	11 (7.5%)	4 (6.1%)	7 (8.6%)	
Bradycardia with poor perfusion	85 (57.8%)	37 (56.1%)	48 (59.3%)	
Duration of CPR (min)	11.0 [5.0, 29.0]	5.0 [3.0, 16.0]	20.0 [8.0, 41.0]	< 0.001
Duration of CPR (min)				< 0.001
< 6	44 (29.9%)	34 (51.5%)	10 (12.3%)	
6–15	39 (26.5%)	14 (21.2%)	25 (30.9%)	
16–35	32 (21.8%)	12 (18.2%)	20 (24.7%)	
> 35	32 (21.8%)	6 (9.1%)	26 (32.1%)	
Pharmacologic interventions during CPR				
Epinephrine	147 (100.0%)	66 (100.0%)	81 (100.0%)	
Minutes to first dose	2.0 [1.0, 3.0]	1.0 [1.0, 2.0]	2.0 [1.0, 3.0]	0.028
Number of doses	2.0 [1.0, 5.0]	2.0 [1.0, 4.0]	3.0 [2.0, 5.0]	< 0.001
Average inter-dose interval^†^	4.5 [3.3, 8.0]	4.0 [3.0, 6.0]	4.8 [3.5, 9.3]	0.072
Calcium	79 (53.7%)	28 (42.4%)	51 (63.0%)	0.020
Sodium bicarbonate	91 (61.9%)	32 (48.5%)	59 (72.8%)	0.004
Pre-epinephrine BP (mmHg)				
Diastolic BP	34.3 [27.9, 45.5]	32.2 [28.5, 41.0]	37.2 [27.6, 46.6]	0.326
Systolic BP	72.1 [52.5, 97.6]	70.7 [52.6, 86.7]	73.3 [52.3, 103.6]	0.327
Adequate diastolic BP^‡^	113 (76.9%)	52 (78.8%)	61 (75.3%)	0.696
Adequate systolic BP^§^	86 (58.5%)	36 (54.5%)	50 (61.7%)	0.399
Change in BP with epinephrine (mmHg)^||^				
Diastolic BP	4.4 [− 1.9, 11.5]	13.6 [7.5, 29.3]	− 1.5 [− 5.0, 1.5]	< 0.001
Systolic BP	11.4 [− 3.6, 25.8]	24.0 [11.5, 38.3]	1.4 [− 9.7, 14.1]	< 0.001

*CPR* cardiopulmonary resuscitation, *PICU* pediatric intensive care unit, *CICU* pediatric cardiac intensive care unit, *PEA* pulseless electrical activity, *VF* ventricular fibrillation, *VT* ventricular tachycardia, *BP* blood pressure

*Weekday is between 7 a.m. and 11 p.m. Monday–Friday; weeknight is after 11 p.m. Monday–Thursday; Weekend is from 11 p.m. on Friday through 7 a.m. on the following Monday

^†^Event-level average interval between epinephrine doses calculated among patients who received at least two doses of epinephrine

^‡^Average diastolic BP prior to first dose of epinephrine of ≥ 25 mmHg for age < 1 year or ≥ 30 mmHg for age ≥ 1 year

^§^Average systolic BP prior to first dose of epinephrine ≥ 60 mmHg for age < 1 year or ≥ 80 mmHg for age ≥ 1 year

^||^Difference in BP from the 30-s data epoch prior to the minute in which the first dose of epinephrine was administered to the average of the four 30-s data epochs following the minute in which epinephrine was administered

Epinephrine responders (patients with ≥ 5 mmHg increase in DBP following the first dose of epinephrine administered during cardiopulmonary resuscitation) and epinephrine non-responders compared using Fisher’s exact test for categorical data and Wilcoxon rank-sum test for continuous data

Epinephrine responders more frequently achieved sustained ROSC than epinephrine non-responders (48/66 [73%] vs. 36/81 [44%]). Return of circulation was achieved via extracorporeal CPR (ECPR) in 10/66 (15%) of epinephrine responders and 39/81 (48%) non-responders. Exploratory survival and functional outcomes did not differ between groups (Table 3). After adjusting for confounders, an increase in DBP ≥ 5 mmHg was associated with higher relative risk of sustained ROSC (1.60 [95% CI 1.21, 2.12]; *p* = 0.001). The sensitivity analysis of other DBP thresholds revealed associations between increases in DBP of ≥ 10 mmHg, ≥ 15 mmHg, and ≥ 20 mmHg and ROSC (Table 4). Additionally, increases in DBP of ≥ 10 mmHg and ≥ 15 mmHg were associated with higher aRR of survival to hospital discharge (≥ 10 mmHg: aRR 1.41 (1.07, 1.86); *p* = 0.013; ≥ 15 mmHg: aRR 1.63 (1.23, 2.17); *p* < 0.001) and survival with favorable neurologic outcome (≥ 10 mmHg: aRR 1.35 (1.01, 1.79); *p* = 0.041; ≥ 15 mmHg: aRR 1.53 (1.13, 2.07); *p* = 0.005).

**Table 3:** Univariate outcomes by epinephrine response

	Overall (*n* = 147)	Epinephrine responders (n = 66)	Epinephrine non-responders (n = 81)	*p*
Immediate event outcome				< 0.001
Sustained ROSC	84 (57%)	48 (73%)	36 (44%)	
Return of Circulation via ECPR	49 (33%)	10 (15%)	39 (48%)	
Death	14 (10%)	8 (12%)	6 (7%)	
Survival to hospital discharge	83 (56%)	39 (59%)	44 (54%)	0.618
Survival to hospital discharge with favorable neurologic outcome*	81 (55%)	37 (56%)	44 (54%)	0.869
Total FSS at hospital discharge^†^	9 [7, 11]	9 [7, 12]	8 [8, 10]	0.280
PCPC at hospital discharge				0.871
1—Normal	39 (27%)	17 (26%)	22 (27%)	
2—Mild disability	21 (14%)	10 (15%)	11 (14%)	
3—Moderate disability	17 (12%)	7 (11%)	10 (12%)	
4—Severe disability	6 (4%)	5 (8%)	1 (1%)	
5—Coma/vegetative state	0 (0%)	0 (0%)	0 (0%)	
6—Death	64 (44%)	27 (41%)	37 (46%)	
Change in FSS from baseline to hospital discharge^†^	2 [0, 3]	1 [0, 4]	2 [0, 3]	0.970
New morbidity^†‡^	27 (33%)	15 (38%)	12 (27%)	0.350

*ROSC* return of spontaneous circulation, *ECPR* extracorporeal cardiopulmonary resuscitation, *FSS* Functional Status Scale, *PCPC* Pediatric Cerebral Performance Category

* Favorable neurologic outcome was defined as no more than moderate disability or no worsening from baseline Pediatric Cerebral Performance Category (PCPC). Baseline PCPC represents subject status prior to the event leading to hospitalization

^†^Includes survivors only

^‡^New morbidity among survivors was defined as a worsening from baseline FSS by 3 points or more

Epinephrine responders (patients with ≥ 5 mmHg increase in DBP following the first dose of epinephrine administered during cardiopulmonary resuscitation) and epinephrine non-responders compared using Fisher’s exact test for categorical data and Wilcoxon rank-sum test for continuous data

**Table 4:** Sensitivity analysis of thresholds of change in DBP and patient outcomes

DBP threshold	Patients meeting threshold (*n* = 147)	ROSC		Survival to hospital discharge		Survival with favorable neurologic outcome*	
		aRR	*p* value	aRR	*p* value	aRR	*p* value
≥ 0 mmHg	98 (66.7%)	1.35 (0.97, 1.87)	0.075	1.10 (0.81, 1.49)	0.549	1.08 (0.79, 1.47)	0.634
≥ 5 mmHg	66 (44.9%)	1.60 (1.21, 2.12)	0.001	1.12 (0.85, 1.47)	0.428	1.09 (0.82, 1.44)	0.560
≥ 10 mmHg	41 (27.9%)	1.59 (1.23, 2.05)	< 0.001	1.41 (1.07, 1.86)	0.013	1.35 (1.01, 1.79)	0.041
≥ 15 mmHg	30 (20.4%)	1.63 (1.27, 2.08)	< 0.001	1.63 (1.23, 2.17)	< 0.001	1.53 (1.13, 2.07)	0.005
≥ 20 mmHg	20 (13.6%)	1.45 (1.08, 1.96)	0.015	1.37 (0.92, 2.04)	0.125	1.35 (0.89, 2.06)	0.158

*DBP* diastolic blood pressure, *ROSC* return of spontaneous circulation, *Arr* adjusted relative risk

*Favorable neurologic outcome was defined as no more than moderate disability or no worsening from baseline Pediatric Cerebral Performance Category (PCPC). Baseline PCPC represents subject status prior to the event leading to hospitalization

Sensitivity analysis exploring potential thresholds for the change in DBP in response to epinephrine and outcomes utilizing Poisson regression model with robust error estimates. Model controlled for a priori specified covariates hypothesized to be associated with both the DBP response and outcomes: initial CPR rhythm (bradycardia and poor perfusion versus pulseless rhythms); illness category (medical cardiac, medical non-cardiac, surgical cardiac, surgical non-cardiac); presence of a vasopressor infusion at the start of CPR; and preexisting pulmonary hypertension

**Fig. 3:**
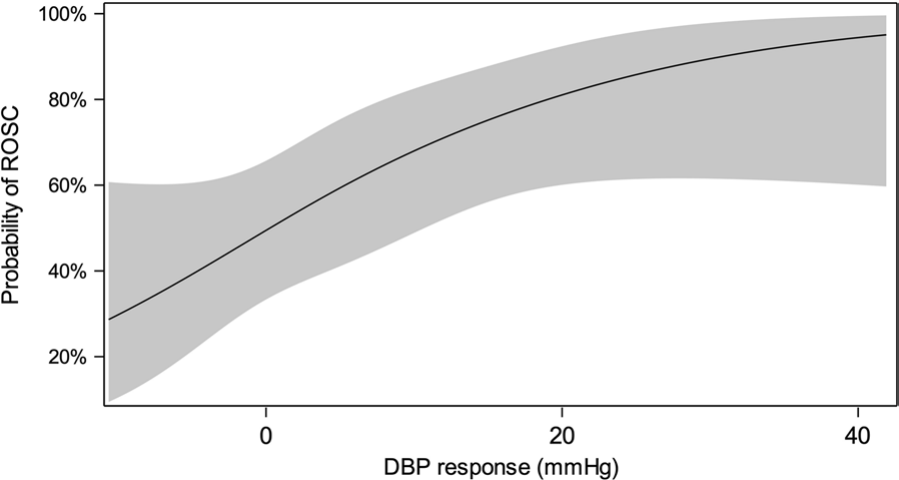
Spline analysis. Spline curve depicting the relationship between change in DBP (diastolic blood pressure) after the first dose of epinephrine and the probability of ROSC (return of spontaneous circulation). Curve based on a logistic regression model controlling for the same a priori covariates as the primary multivariate analysis (initial CPR rhythm (bradycardia and poor perfusion versus pulseless rhythms); illness category (medical cardiac, medical non-cardiac, surgical cardiac, surgical non-cardiac); presence of a vasopressor infusion at the start of CPR; and preexisting pulmonary hypertension). Shaded area represents 95% confidence interval. The optimal cut point for discriminating patients with and without ROSC, based on receiver operating characteristic curve analysis, was an increase in DBP of 4.4 mmHg

Figure 3 is the spline curve depicting the relationship between change in DBP after the first dose of epinephrine and the probability of ROSC. The optimal cut point for discriminating patients with and without ROSC was an increase in DBP of 4.4 mmHg. Additional file 3: Table S3 and Additional file 4: Table S4 compare patient and arrest characteristics between patients with and without ROSC.

## Discussion

The data from this study support our hypotheses that the physiologic response to epinephrine during pediatric CPR is variable among patients and is associated with event outcome. Specifically, patients meeting the threshold increase in DBP of ≥ 5 mmHg after epinephrine administration had 60% higher likelihood of ROSC compared to those without a hemodynamic response of this magnitude. This finding was robust through additional higher DBP thresholds of ≥ 10 mmHg, ≥ 15 mmHg, and ≥ 20 mmHg. Moreover, thresholds of ≥ 10 mmHg and ≥ 15 mmHg were associated with higher adjusted relative risks of survival to hospital discharge and survival with favorable neurologic outcome. To our knowledge, this is the first clinical study to demonstrate the association of the hemodynamic response to epinephrine during CPR with outcomes and the first dedicated study to describe the hemodynamic response to epinephrine during CPR in children.

The change in DBP in response to the first dose of epinephrine during CPR varied widely among patients in this study with an interquartile range of − 1.9 mmHg to 11.5 mmHg. Variable responses to adrenergic agonists have been characterized in experimental studies and in other disease states and are likely due to a host of factors [14–17, 26–28], including genetic polymorphisms in adrenergic receptors [14, 27, 29–32]. Such genetic variation and other aspects of adrenergic receptor expression and physiology may contribute to the differences observed in epinephrine response. Alpha-1 receptor physiology may be implicated as epinephrine’s principal role during cardiac arrest to cause vasoconstriction via alpha-1 receptor agonism and thereby increase CoPP. Importantly though, 58% of children in our study received CPR for an initial rhythm of bradycardia with poor perfusion rather than pulseless IHCA. Since these patients still have some degree of native myocardial function, epinephrine serves the dual role of augmenting systemic vascular resistance as well as serving as an inotrope through beta-1 receptor-mediated effects. Thus, differential beta-adrenergic effects may also play a role in our findings [31, 33–35].

Demographic and phenotypic differences among patients likely contribute to the variability in epinephrine responses. Only 13 of 48 (27%) children under one month of age were classified as epinephrine responders compared to 10 of 11 (91%) children older than 12 years. Further, fewer than 40% of patients with primary cardiac illness categories or congenital heart disease were epinephrine responders. These data suggest that younger patients and children with heart disease may be intrinsically less likely to respond, potentially due to differences in vascular tone and reactivity, severity of myocardial injury, immature myocardial responsiveness to adrenergic medications, or co-administration of inodilators and other medications. We hypothesized that patients requiring vasoactive infusions at the time of arrest would be less likely to respond to epinephrine as this could represent a population of patients progressing to IHCA due to catecholamine-refractory shock for whom additional catecholamines during CPR could be less efficacious. However, we did not observe differences between responder groups in terms of the presence of vasoactive agents, the pre-arrest vasoactive-inotrope score, or the frequency of hypotension as the immediate cause of arrest. We also chose pulmonary hypertension as an a priori covariate in our multivariable models due to laboratory data demonstrating inadequate intra-arrest blood pressures despite epinephrine administration in animals with pulmonary hypertension-associated cardiac arrest [36]; however, the prevalence of pulmonary hypertension was not different between groups. The quality of CPR and other intra-arrest therapies or interventions also may impact the response to epinephrine. As CPR quality was primarily measured by patient physiology in the ICU-RESUS trial, chest compression mechanics data were not widely available for this cohort. Thus, we cannot fully account for potential differences in CPR quality. Of note, the absolute DBP prior to epinephrine, a physiologic metric of CPR quality, was similar between groups. Overall, the factors influencing the physiologic response to epinephrine during CPR are likely complex and further investigation is merited to delineate which patients are most likely to derive physiologic benefit from epinephrine administration during CPR.

We examined ROSC as our primary outcome because it is the most proximate CPR outcome that would reflect intra-CPR physiology without the confounding influence of the post-arrest period. In addition to more commonly achieving ROSC (aRR 1.60 [95% CI 1.21, 2.12]), responders had significantly shorter CPR duration (5 [3, 16] vs. 20 [8, 41] minutes), as we expected based on the relationship between attaining adequate DBP and achieving ROSC [2, 22]. These findings are also consistent with a large animal laboratory study in a pediatric model of cardiac arrest in which the magnitude of DBP change after the first dose of epinephrine was higher among survivors than non-survivors and correlated with time to ROSC [11]. The validity of our findings is supported by the sensitivity analysis revealing that thresholds of DBP response higher than 5 mmHg were also associated with ROSC with adjusted relative risks of similar magnitude. While the relative risks of ROSC were similar among these various thresholds, the spline curve (Fig. 3) exploring the relationship between change in DBP and ROSC suggests a possible “dose–response effect” with the probability of ROSC continuing to increase well beyond the a priori threshold of 5 mmHg. Importantly, the ideal threshold for change in DBP may differ according to patient age or other characteristics and this merits further investigation in larger datasets.

Some of these higher epinephrine response thresholds (≥ 10 mmHg and ≥ 15 mmHg) were also associated with survival to hospital discharge and survival with favorable neurologic outcome, suggesting that patients with a particularly robust hemodynamic response to epinephrine may benefit beyond the CPR event itself. These higher DBPs and resultant mean arterial pressures may have resulted in sufficient myocardial and cerebral blood flow to mitigate intra-arrest myocardial and cerebral injury, thus leading to superior survival rates and neurologic outcomes. We did not detect differences in these longer-term outcomes with our primary exposure (≥ 5 mmHg), presumably in part due to the relatively high use of ECPR in this non-responder population. Though only 44% of epinephrine non-responders achieved ROSC, an additional 48% achieved return of circulation via ECPR, compared to 73% with ROSC and 15% with ECPR in the responder group. In other words, nearly half of the patients without a ≥ 5 mmHg physiologic response to epinephrine failed to achieve ROSC but received ECPR as a rescue therapy and therefore had the potential to survive to discharge. Additionally, though they frequently required ECPR and had considerably longer median CPR durations, the similar rate of neurologically intact survival in the non-responder group also likely reflects the high percentage of surgical cardiac patients in this group (49%), patients who frequently have acute reversible cardiac dysfunction and relatively good IHCA outcomes [37].

The findings of this study have potential implications on cardiac arrest care at the bedside. Resuscitation guidelines advocate for targeting DBP during CPR but lack specificity on how to do so [4]. Observing a noticeable increase in DBP in response to epinephrine with a subsequent decline in DBP may justify an “early” subsequent dose in an attempt to achieve the same effect, as is suggested by animal studies of shorter intervals between epinephrine doses with hemodynamic-directed CPR [38, 39] and a clinical study in which more frequent epinephrine was associated with superior outcomes [40]. Conversely, failure to achieve a hemodynamic response to epinephrine may be reason to avoid further administration of an ineffective therapy. Preclinical data suggest that when epinephrine increases systemic blood pressures, cerebral blood flow and oxygenation similarly increase [9, 12, 41, 42]. In patients who fail to respond in terms of systemic hemodynamics, deleterious effects of epinephrine may predominate [43, 44] and this may contribute to the failure of epinephrine to improve neurologic outcomes in some studies [6]. However, as this study only addressed the response to the first dose of epinephrine, it is unknown whether lack of response to the first dose predicts a lack of response to subsequent doses as there may be both static and dynamic factors influencing the response. Thus, lack of response to epinephrine should likely serve as an indication to redouble efforts to identify and treat the underlying cause of arrest or other pathophysiologic processes preventing a response to epinephrine. It may also serve as an indication to provide alternative therapies to epinephrine. In an animal model of pediatric IHCA, a proportion of subjects that failed to reach BP goals after two doses of epinephrine successfully reached those goals after a dose of vasopressin, suggesting a potential role for vasopressin as hemodynamic rescue therapy in pediatric CPR [10]. Finally, knowledge that patients without robust DBP responses to epinephrine early in arrest are likely to require prolonged CPR and less likely to achieve ROSC may provide justification for early activation of ECPR systems, an assertion supported by the high utilization of ECPR in the non-responders in this study.

The limitations of this study are important to consider in interpreting its findings. First, the observational study design precludes our ability to determine causative relationships between physiologic observations and outcomes. However, limitations in study design are mitigated by the fact that this secondary study consisted entirely of prospectively collected data from the ICU-RESUS trial and the analysis itself was designed during trial enrollment without review of the data. Additionally, robust physiologic waveform review and analysis methods safeguarded against the inclusion of spurious data and ensured that the physiologic data analyzed represents only periods of CPR. Second, this study was conducted at large academic referral centers in the USA—the generalizability of these findings requires broader investigations. Third, we cannot guarantee the accuracy with which the timing of the first epinephrine administration was recorded and we did not record timing of subsequent epinephrine doses or other potentially relevant interventions during CPR. Fourth, this study did not examine the mechanisms by which the DBP response to epinephrine varies among patients. Though we were able to report on demographic and clinical features of responders and non-responders, more in-depth elucidation of these phenotypes and genotypic characterization of these patients will be necessary in future work. Fifth, our definition of epinephrine responders required invasive arterial BP monitoring and evaluable pre- and post-epinephrine BP data, and thus may not be easily generalizable for patients without invasive BP monitoring. Children included in this study differed from those who were excluded due to absent or insufficient BP data in terms of several pre-arrest and arrest characteristics. However, close to half of children with IHCA in the ICU have invasive BP monitoring in place [45] and this is precisely the group of patients that may benefit from physiologic-directed CPR. Differences in DBP measurement techniques between research studies and bedside monitors deserve further study to enhance the applicability of these findings. Finally, though the observation of superior event outcomes in epinephrine responders is clinically novel, interventions to improve outcomes among non-responders require further evaluation.

## Conclusions

In this prospective multicenter observational study of pediatric IHCA, children with at least a 5 mmHg increase in DBP after administration of epinephrine were more likely to achieve sustained ROSC than those without such a DBP response.

## Supplementary Information

**Additional file 1. Supplemental Table 1.** Patient Characteristics of Included Versus Excluded Subjects.

**Additional file 2. Supplemental Table 2.** Cardiac Arrest Event Characteristics of Included Versus Excluded Subjects.

**Additional file 3. Supplemental Table 3.** Patient Characteristics between Patients with and without Return of Spontaneous Circulation.

**Additional file 4. Supplemental Table 4.** Cardiac Arrest Event Characteristics between Patients with and without Return of Spontaneous Circulation.

## Data Availability

Data from the ICU-RESUS clinical trial, including deidentified patient data and the data dictionary, will be made available upon reasonable request to the corresponding author of the parent trial (suttonr@chop.edu).
