# Supplementary material for: The physiologic response to epinephrine and pediatric cardiopulmonary resuscitation outcomes
Source: Crit Care. 2023 Mar 13;27:105. doi: 10.1186/s13054-023-04399-5 (PMC10012560; doi:10.1186/s13054-023-04399-5)
Supplement: Supplementary file 1 — Additional file 1. Supplemental Table 1. Patient Characteristics of Included Versus Excluded Subjects. [file 13054_2023_4399_MOESM1_ESM.docx]

**Supplemental Table 1.** Patient Characteristics of Included Versus Excluded Subjects

|  | **Overall**  **(n=894)** | **Final Cohort**  **(n=147)** | **Excluded Subjects**  **(n=747)** | ***p*** |
| --- | --- | --- | --- | --- |
| **Demographics** |  |  |  |  |
| Age (years) | 0.6 [0.2,4.5] | 0.3 [0.0,1.7] | 0.8 [0.2,5.8] | <.001 |
| Age |  |  |  | <.001 |
| <1 month | 150 (16.8%) | 48 (32.7%) | 102 (13.7%) |  |
| 1 month-<1 year | 359 (40.2%) | 57 (38.8%) | 302 (40.4%) |  |
| 1 year-<12 years | 277 (31.0%) | 31 (21.1%) | 246 (32.9%) |  |
| >12 years | 108 (12.1%) | 11 (7.5%) | 97 (13.0%) |  |
| **Male** | 477 (53.4%) | 71 (48.3%) | 406 (54.4%) | 0.205 |
| **Race** |  |  |  | 0.383 |
| White | 419 (46.9%) | 73 (49.7%) | 346 (46.3%) |  |
| Black or African American | 226 (25.3%) | 31 (21.1%) | 195 (26.1%) |  |
| Other | 51 (5.7%) | 10 (6.8%) | 41 (5.5%) |  |
| Unknown or Not Reported | 198 (22.1%) | 33 (22.4%) | 165 (22.1%) |  |
| **Preexisting Conditions** |  |  |  |  |
| Respiratory insufficiency | 769 (86.0%) | 123 (83.7%) | 646 (86.5%) | 0.364 |
| Hypotension | 620 (69.4%) | 113 (76.9%) | 507 (67.9%) | 0.031 |
| Congenital heart disease | 528 (59.1%) | 105 (71.4%) | 423 (56.6%) | <.001 |
| Pulmonary hypertension | 152 (17.0%) | 24 (16.3%) | 128 (17.1%) | 0.905 |
| Sepsis | 160 (17.9%) | 18 (12.2%) | 142 (19.0%) | 0.059 |
| Renal insufficiency | 133 (14.9%) | 15 (10.2%) | 118 (15.8%) | 0.099 |
| Congestive heart failure | 120 (13.4%) | 14 (9.5%) | 106 (14.2%) | 0.146 |
| Pneumonia | 109 (12.2%) | 14 (9.5%) | 95 (12.7%) | 0.335 |
| Malignancy | 48 (5.4%) | 7 (4.8%) | 41 (5.5%) | 0.843 |
| Trauma | 29 (3.2%) | 1 (0.7%) | 28 (3.7%) | 0.070 |
| **Pre-event Characteristics** |  |  |  |  |
| Illness category |  |  |  | <.001 |
| Medical cardiac | 207 (23.2%) | 38 (25.9%) | 169 (22.6%) |  |
| Surgical cardiac | 325 (36.4%) | 69 (46.9%) | 256 (34.3%) |  |
| Non-cardiac | 362 (40.5%) | 40 (27.2%) | 322 (43.1%) |  |
| Baseline PCPC score* |  |  |  | 0.003 |
| 1 - Normal | 544 (60.9%) | 103 (70.1%) | 441 (59.0%) |  |
| 2 - Mild disability | 167 (18.7%) | 28 (19.0%) | 139 (18.6%) |  |
| 3 - Moderate disability | 89 (10.0%) | 9 (6.1%) | 80 (10.7%) |  |
| 4 - Severe disability | 87 (9.7%) | 7 (4.8%) | 80 (10.7%) |  |
| 5 - Coma | 7 (0.8%) | 0 (0.0%) | 7 (0.9%) |  |
| Baseline FSS* | 6.0 [6.0,10.0] | 6.0 [6.0,8.0] | 6.0 [6.0,10.0] | 0.006 |
| PRISM^†^ | 5.0 [0.0,11.0] | 7.0 [2.0,12.0] | 5.0 [0.0,11.0] | 0.014 |
| Vasoactive inotropic score^‡^ | 0.0 [0.0,8.0] | 4.0 [0.0,10.0] | 0.0 [0.0,7.5] | <.001 |
| Vasopressors used^‡^ |  |  |  |  |
| Dopamine | 118 (13.2%) | 30 (20.4%) | 88 (11.8%) | 0.007 |
| Dobutamine | 10 (1.1%) | 1 (0.7%) | 9 (1.2%) | 1.000 |
| Nitroprusside | 10 (1.1%) | 3 (2.0%) | 7 (0.9%) | 0.218 |
| Milrinone | 215 (24.0%) | 48 (32.7%) | 167 (22.4%) | 0.011 |
| Epinephrine | 249 (27.9%) | 55 (37.4%) | 194 (26.0%) | 0.006 |
| Norepinephrine | 58 (6.5%) | 6 (4.1%) | 52 (7.0%) | 0.270 |
| Phenylephrine | 3 (0.3%) | 0 (0.0%) | 3 (0.4%) | 1.000 |
| Vasopressin | 51 (5.7%) | 6 (4.1%) | 45 (6.0%) | 0.439 |

PRISM = Pediatric RISk of Mortality; PCPC = Pediatric Cerebral Performance Category; FSS = Functional Status Scale.
*Baseline PCPC and FSS represent subject status prior to the event leading to hospitalization.

^†^PRISM was evaluated 2 - 6 hours prior to the event.
^‡^Vasoactive inotropic score and vasopressors used were evaluated 2 hours prior to the event.

Characteristics among subjects included in final cohort and subjects who received epinephrine but were excluded from the final cohort compared using Fisher’s exact test for categorical data and Wilcoxon rank-sum test for continuous data.
