# Supplementary material for: The physiologic response to epinephrine and pediatric cardiopulmonary resuscitation outcomes
Source: Crit Care. 2023 Mar 13;27:105. doi: 10.1186/s13054-023-04399-5 (PMC10012560; doi:10.1186/s13054-023-04399-5)
Supplement: Supplementary file 2 — Additional file 2. Supplemental Table 2. Cardiac Arrest Event Characteristics of Included Versus Excluded Subjects. [file 13054_2023_4399_MOESM2_ESM.docx]

**Supplemental Table 2.** Cardiac Arrest Event Characteristics of Included Versus Excluded Subjects

|  | **Overall**  **(n=894)** | **Final Cohort**  **(n=147)** | **Excluded Subjects**  **(n=747)** | ***p*** |
| --- | --- | --- | --- | --- |
| **Location of CPR Event** |  |  |  | <.001 |
| PICU | 420 (47.0%) | 46 (31.3%) | 374 (50.1%) |  |
| CICU | 474 (53.0%) | 101 (68.7%) | 373 (49.9%) |  |
| **Interventions in Place** |  |  |  |  |
| Central venous catheter | 631 (70.6%) | 119 (81.0%) | 512 (68.5%) | 0.002 |
| Vasoactive infusion | 515 (57.6%) | 102 (69.4%) | 413 (55.3%) | 0.002 |
| Invasive mechanical ventilation | 656 (73.4%) | 120 (81.6%) | 536 (71.8%) | 0.014 |
| Non-invasive ventilation | 161 (18.0%) | 15 (10.2%) | 146 (19.5%) | 0.007 |
| **Immediate Cause(s) of Arrest** |  |  |  |  |
| Arrhythmia | 149 (16.7%) | 23 (15.6%) | 126 (16.9%) | 0.809 |
| Cyanosis without respiratory decompensation | 42 (4.7%) | 7 (4.8%) | 35 (4.7%) | 1.000 |
| Hypotension | 545 (61.0%) | 101 (68.7%) | 444 (59.4%) | 0.042 |
| Respiratory decompensation | 456 (51.0%) | 67 (45.6%) | 389 (52.1%) | 0.176 |
| **Timing of CPR Event^*^** |  |  |  | 0.004 |
| Weekday | 465 (52.0%) | 90 (61.2%) | 375 (50.2%) |  |
| Weeknight | 181 (20.2%) | 32 (21.8%) | 149 (19.9%) |  |
| Weekend | 248 (27.7%) | 25 (17.0%) | 223 (29.9%) |  |
| **First Documented Rhythm** |  |  |  | 0.141 |
| Asystole / PEA | 372 (41.6%) | 51 (34.7%) | 321 (43.0%) |  |
| VF / pulseless VT | 70 (7.8%) | 11 (7.5%) | 59 (7.9%) |  |
| Bradycardia with poor perfusion | 452 (50.6%) | 85 (57.8%) | 367 (49.1%) |  |
| **Duration of CPR (minutes)** | 10.0 [4.0,31.0] | 11.0 [5.0,29.0] | 10.0 [4.0,31.0] | 0.356 |
| **Duration of CPR (minutes)** |  |  |  | 0.727 |
| <6 | 302 (33.8%) | 44 (29.9%) | 258 (34.5%) |  |
| 6-15 | 216 (24.2%) | 39 (26.5%) | 177 (23.7%) |  |
| 16-35 | 188 (21.0%) | 32 (21.8%) | 156 (20.9%) |  |
| >35 | 188 (21.0%) | 32 (21.8%) | 156 (20.9%) |  |
| **Pharmacologic Interventions during CPR** |  |  |  |  |
| Epinephrine | 894 (100.0%) | 147 (100.0%) | 747 (100.0%) |  |
| Minutes to first dose | 1.0 [0.0,2.0] | 2.0 [1.0,3.0] | 1.0 [0.0,2.0] | <.001 |
| Number of doses | 3.0 [1.0,6.0] | 2.0 [1.0,5.0] | 3.0 [1.0,7.0] | 0.398 |
| Average inter-dose interval^†^ | 4.1 [3.1,6.0] | 4.5 [3.3,8.0] | 4.0 [3.0,6.0] | 0.038 |
| Calcium | 457 (51.1%) | 79 (53.7%) | 378 (50.6%) | 0.528 |
| Sodium bicarbonate | 530 (59.3%) | 91 (61.9%) | 439 (58.8%) | 0.521 |
| **Pre-Epinephrine BP (mmHg)** |  |  |  |  |
| Diastolic BP | 35.0 [27.4,46.2] | 34.3 [27.9,45.5] | 36.8 [26.2,50.6] | 0.459 |
| Systolic BP | 72.4 [53.2,99.6] | 72.1 [52.5,97.6] | 72.4 [59.1,106.0] | 0.593 |
| Adequate Diastolic BP^‡^ | 144 (16.1%) | 113 (76.9%) | 31 (4.1%) | 0.685 |
| Adequate Systolic BP^§^ | 110 (12.3%) | 86 (58.5%) | 24 (3.2%) | 1.000 |

CPR = cardiopulmonary resuscitation; PICU = pediatric intensive care unit; CICU = pediatric cardiac intensive care unit; PEA = pulseless electrical activity; VF = ventricular fibrillation; VT = ventricular tachycardia; BP = blood pressure.
* Weekday is between 7 AM and 11 PM Monday - Friday; weeknight is after 11 PM Monday - Thursday; Weekend is from 11 PM on Friday through 7 AM on the following Monday.

^†^Event-level average interval between epinephrine doses calculated among patients who received at least two doses of epinephrine.
^‡^Average diastolic BP prior to first dose of epinephrine of ≥25 mmHg for age <1 year or ≥30 mmHg for age ≥1 year.
^§^Average systolic BP prior to first dose of epinephrine ≥60 mmHg for age <1 year or ≥80 mmHg for age ≥1 year.

Characteristics among subjects included in final cohort and subjects who received epinephrine but were excluded from the final cohort compared using Fisher’s exact test for categorical data and Wilcoxon rank-sum test for continuous data.
