# Supplementary material for: The physiologic response to epinephrine and pediatric cardiopulmonary resuscitation outcomes
Source: Crit Care. 2023 Mar 13;27:105. doi: 10.1186/s13054-023-04399-5 (PMC10012560; doi:10.1186/s13054-023-04399-5)
Supplement: Supplementary file 3 — Additional file 3. Supplemental Table 3. Patient Characteristics between Patients with and without Return of Spontaneous Circulation. [file 13054_2023_4399_MOESM3_ESM.docx]

**Supplemental Table 3.** Patient Characteristics between Patients with and without Return of Spontaneous Circulation

|  | **Overall**  **(n=147)** | **ROSC**  **(n=84)** | **No ROSC**  **(n=63)** | ***p*** |
| --- | --- | --- | --- | --- |
| **Demographics** |  |  |  |  |
| Age (years) | 0.3 [0.0,1.7] | 0.3 [0.1,1.5] | 0.2 [0.0,2.2] | 0.368 |
| Age |  |  |  | 0.426 |
| <1 month | 48 (32.7%) | 22 (26.2%) | 26 (41.3%) |  |
| 1 month-<1 year | 57 (38.8%) | 39 (46.4%) | 18 (28.6%) |  |
| 1 year-<12 years | 31 (21.1%) | 19 (22.6%) | 12 (19.0%) |  |
| >12 years | 11 (7.5%) | 4 (4.8%) | 7 (11.1%) |  |
| **Male** | 71 (48.3%) | 38 (45.2%) | 33 (52.4%) | 0.409 |
| **Race** |  |  |  | 0.312 |
| White | 73 (49.7%) | 39 (46.4%) | 34 (54.0%) |  |
| Black or African American | 31 (21.1%) | 17 (20.2%) | 14 (22.2%) |  |
| Other | 10 (6.8%) | 8 (9.5%) | 2 (3.2%) |  |
| Unknown or Not Reported | 33 (22.4%) | 20 (23.8%) | 13 (20.6%) |  |
| **Preexisting Conditions** |  |  |  |  |
| Respiratory insufficiency | 123 (83.7%) | 74 (88.1%) | 49 (77.8%) | 0.116 |
| Hypotension | 113 (76.9%) | 59 (70.2%) | 54 (85.7%) | 0.031 |
| Congestive heart failure | 14 (9.5%) | 5 (6.0%) | 9 (14.3%) | 0.098 |
| Pneumonia | 14 (9.5%) | 10 (11.9%) | 4 (6.3%) | 0.395 |
| Sepsis | 18 (12.2%) | 8 (9.5%) | 10 (15.9%) | 0.311 |
| Renal insufficiency | 15 (10.2%) | 6 (7.1%) | 9 (14.3%) | 0.178 |
| Malignancy | 7 (4.8%) | 3 (3.6%) | 4 (6.3%) | 0.462 |
| Congenital heart disease | 105 (71.4%) | 59 (70.2%) | 46 (73.0%) | 0.854 |
| Trauma | 1 (0.7%) | 1 (1.2%) | 0 (0.0%) | 1.000 |
| Pulmonary hypertension | 24 (16.3%) | 13 (15.5%) | 11 (17.5%) | 0.823 |
| **Pre-event Characteristics** |  |  |  |  |
| Illness category |  |  |  | 0.131 |
| Medical cardiac | 38 (25.9%) | 17 (20.2%) | 21 (33.3%) |  |
| Surgical cardiac | 69 (46.9%) | 40 (47.6%) | 29 (46.0%) |  |
| Non-cardiac | 40 (27.2%) | 27 (32.1%) | 13 (20.6%) |  |
| Baseline PCPC score* |  |  |  | 0.266 |
| 1 - Normal | 103 (70.1%) | 56 (66.7%) | 47 (74.6%) |  |
| 2 - Mild disability | 28 (19.0%) | 17 (20.2%) | 11 (17.5%) |  |
| 3 - Moderate disability | 9 (6.1%) | 6 (7.1%) | 3 (4.8%) |  |
| 4 - Severe disability | 7 (4.8%) | 5 (6.0%) | 2 (3.2%) |  |
| Baseline FSS* | 6.0 [6.0,8.0] | 6.0 [6.0,9.0] | 6.0 [6.0,8.0] | 0.186 |
| PRISM^†^ | 7.0 [2.0,12.0] | 5.5 [1.0,10.0] | 8.0 [3.0,15.0] | 0.011 |
| Vasoactive inotropic score^‡^ | 4.0 [0.0,10.0] | 1.3 [0.0,6.9] | 7.0 [2.0,15.0] | <.001 |
| Vasopressors used^‡^ |  |  |  |  |
| Dopamine | 30 (20.4%) | 15 (17.9%) | 15 (23.8%) | 0.413 |
| Dobutamine | 1 (0.7%) | 0 (0.0%) | 1 (1.6%) | 0.429 |
| Nitroprusside | 3 (2.0%) | 1 (1.2%) | 2 (3.2%) | 0.577 |
| Milrinone | 48 (32.7%) | 23 (27.4%) | 25 (39.7%) | 0.155 |
| Epinephrine | 55 (37.4%) | 23 (27.4%) | 32 (50.8%) | 0.006 |
| Norepinephrine | 6 (4.1%) | 2 (2.4%) | 4 (6.3%) | 0.402 |
| Vasopressin | 6 (4.1%) | 2 (2.4%) | 4 (6.3%) | 0.402 |

PRISM = Pediatric RISk of Mortality; PCPC = Pediatric Cerebral Performance Category; FSS = Functional Status Scale.
*Baseline PCPC and FSS represent subject status prior to the event leading to hospitalization.

^†^PRISM was evaluated 2 - 6 hours prior to the event.
^‡^Vasoactive inotropic score and vasopressors used were evaluated 2 hours prior to the event.

Patients with and without return of spontaneous circulation compared using Fisher’s exact test for categorical data and Wilcoxon rank-sum test for continuous data.
