# Supplementary material for: The physiologic response to epinephrine and pediatric cardiopulmonary resuscitation outcomes
Source: Crit Care. 2023 Mar 13;27:105. doi: 10.1186/s13054-023-04399-5 (PMC10012560; doi:10.1186/s13054-023-04399-5)
Supplement: Supplementary file 4 — Additional file 4. Supplemental Table 4. Cardiac Arrest Event Characteristics between Patients with and without Return of Spontaneous Circulation. [file 13054_2023_4399_MOESM4_ESM.docx]

**Supplemental Table 4.** Cardiac Arrest Event Characteristics between Patients with and without Return of Spontaneous Circulation

|  | **Overall**  **(n=147)** | **ROSC**  **(n=84)** | **No ROSC**  **(n=63)** | ***p*** |
| --- | --- | --- | --- | --- |
| **Location of CPR Event** |  |  |  | 0.372 |
| PICU | 46 (31.3%) | 29 (34.5%) | 17 (27.0%) |  |
| CICU | 101 (68.7%) | 55 (65.5%) | 46 (73.0%) |  |
| **Interventions in Place** |  |  |  |  |
| Central venous catheter | 119 (81.0%) | 62 (73.8%) | 57 (90.5%) | 0.011 |
| Vasoactive infusion | 102 (69.4%) | 48 (57.1%) | 54 (85.7%) | <.001 |
| Invasive mechanical ventilation | 120 (81.6%) | 63 (75.0%) | 57 (90.5%) | 0.018 |
| Non-invasive ventilation | 15 (10.2%) | 12 (14.3%) | 3 (4.8%) | 0.096 |
| **Immediate Cause(s) of Arrest** |  |  |  |  |
| Arrhythmia | 23 (15.6%) | 13 (15.5%) | 10 (15.9%) | 1.000 |
| Cyanosis without respiratory decompensation | 7 (4.8%) | 3 (3.6%) | 4 (6.3%) | 0.462 |
| Hypotension | 101 (68.7%) | 50 (59.5%) | 51 (81.0%) | 0.007 |
| Respiratory decompensation | 67 (45.6%) | 48 (57.1%) | 19 (30.2%) | 0.001 |
| **Timing of CPR Event^*^** |  |  |  | 0.395 |
| Weekday | 90 (61.2%) | 51 (60.7%) | 39 (61.9%) |  |
| Weeknight | 32 (21.8%) | 16 (19.0%) | 16 (25.4%) |  |
| Weekend | 25 (17.0%) | 17 (20.2%) | 8 (12.7%) |  |
| **First Documented Rhythm** |  |  |  | 0.161 |
| Asystole / PEA | 51 (34.7%) | 24 (28.6%) | 27 (42.9%) |  |
| VF / pulseless VT | 11 (7.5%) | 6 (7.1%) | 5 (7.9%) |  |
| Bradycardia with poor perfusion | 85 (57.8%) | 54 (64.3%) | 31 (49.2%) |  |
| **Duration of CPR (minutes)** | 11.0 [5.0,29.0] | 6.0 [3.5,10.0] | 38.0 [20.0,54.0] | <.001 |
| **Duration of CPR (minutes)** |  |  |  | <.001 |
| <6 | 44 (29.9%) | 41 (48.8%) | 3 (4.8%) |  |
| 6-15 | 39 (26.5%) | 31 (36.9%) | 8 (12.7%) |  |
| 16-35 | 32 (21.8%) | 12 (14.3%) | 20 (31.7%) |  |
| >35 | 32 (21.8%) | 0 (0.0%) | 32 (50.8%) |  |
| **Pharmacologic Interventions during CPR** |  |  |  |  |
| Epinephrine | 147 (100.0%) | 84 (100.0%) | 63 (100.0%) |  |
| Minutes to first dose | 2.0 [1.0,3.0] | 1.0 [1.0,2.0] | 2.0 [1.0,3.0] | 0.025 |
| Number of doses | 2.0 [1.0,5.0] | 2.0 [1.0,3.0] | 5.0 [3.0,10.0] | <.001 |
| Average inter-dose interval^†^ | 4.5 [3.3,8.0] | 4.5 [3.0,6.0] | 4.5 [3.4,10.0] | 0.083 |
| Calcium | 79 (53.7%) | 34 (40.5%) | 45 (71.4%) | <.001 |
| Sodium bicarbonate | 91 (61.9%) | 40 (47.6%) | 51 (81.0%) | <.001 |
| **Pre-Epinephrine BP (mmHg)** |  |  |  |  |
| Diastolic BP | 34.3 [27.9,45.5] | 35.2 [29.1,46.7] | 33.7 [24.3,42.4] | 0.133 |
| Systolic BP | 72.1 [52.5,97.6] | 73.8 [56.2,100.4] | 71.0 [50.5,90.3] | 0.347 |
| Adequate Diastolic BP^‡^ | 113 (76.9%) | 69 (82.1%) | 44 (69.8%) | 0.113 |
| Adequate Systolic BP^§^ | 86 (58.5%) | 55 (65.5%) | 31 (49.2%) | 0.064 |
| **Change in BP with Epinephrine (mmHg)^\|\|^** |  |  |  |  |
| Diastolic BP | 4.4 [-1.9,11.5] | 6.4 [-1.0,16.8] | 0.6 [-3.5,5.7] | <.001 |
| Systolic BP | 11.4 [-3.6,25.8] | 16.2 [-0.8,32.7] | 7.2 [-7.1,15.1] | 0.001 |

ROSC = return of spontaneous circulation; CPR = cardiopulmonary resuscitation; PICU = pediatric intensive care unit; CICU = pediatric cardiac intensive care unit; PEA = pulseless electrical activity; VF = ventricular fibrillation; VT = ventricular tachycardia; BP = blood pressure.
* Weekday is between 7 AM and 11 PM Monday - Friday; weeknight is after 11 PM Monday - Thursday; Weekend is from 11 PM on Friday through 7 AM on the following Monday.

^†^Event-level average interval between epinephrine doses calculated among patients who received at least two doses of epinephrine.
^‡^Average diastolic BP prior to first dose of epinephrine of ≥25 mmHg for age <1 year or ≥30 mmHg for age ≥1 year.
^§^Average systolic BP prior to first dose of epinephrine ≥60 mmHg for age <1 year or ≥80 mmHg for age ≥1 year.

^||^Difference in BP from the 30-second data epoch prior to the minute in which the first dose of epinephrine was administered to the average of the four 30-second data epochs following the minute in which epinephrine was administered.

Patients with and without return of spontaneous circulation compared using Fisher’s exact test for categorical data and Wilcoxon rank-sum test for continuous data.
